# Integrating Synchronized Nasal Intermittent Positive Pressure Ventilation With Less‐Invasive Surfactant Administration: Current Evidence and Evolving Practice in Preterm Neonatal Care

**DOI:** 10.1002/pdi3.70068

**Published:** 2026-09-30

**Authors:** Aniket P. Singh, Raghuram Mallaiah

**Affiliations:** ^1^ Department of Neonatology The Women's Hospital New Delhi India

## Abstract

The objective of this study was to summarize current evidence on the combined use of synchronized nasal intermittent positive pressure ventilation (sNIPPV) and less‐invasive surfactant administration (LISA) in the management of respiratory distress syndrome (RDS) in preterm infants. We conducted a narrative review of randomized controlled trials, comparative‐effectiveness studies, meta‐analyses, the 2022 European Consensus Guidelines on RDS, and recent clinical cohorts evaluating sNIPPV, LISA, and their combined application. Studies suggest that sNIPPV provides superior extubation tolerance and decreases apnea frequency compared with standard continuous positive airway pressure (CPAP). Compared with the intubation, surfactant administration, and extubation (INSURE) approach, LISA lowers early mechanical ventilation needs and decreases bronchopulmonary dysplasia (BPD) rates. When applied together, the two strategies appear to improve procedural stability and decrease the likelihood of re‐intubation, and may thus contribute to lower BPD rates due to their complementary physiologic effects. The sNIPPV–LISA combination is physiologically complementary and clinically promising. Current evidence supports a reduced rate of respiratory failure and a trend toward reduced BPD compared with CPAP and the INSURE technique of surfactant administration. Larger randomized trials are required to determine its effects on long‐term outcomes.

## 1 Introduction

Respiratory distress syndrome (RDS) remains a leading cause of morbidity and mortality in preterm infants, particularly those born before 32 weeks of gestation. Both synchronized nasal intermittent positive pressure ventilation (sNIPPV) and less‐invasive surfactant administration (LISA) have emerged as major noninvasive strategies designed to reduce the need for mechanical ventilation and ventilator‐associated lung injury. According to the 2022 European Consensus Guidelines on the Management of RDS [1], early application of continuous positive airway pressure (CPAP) or nasal intermittent positive pressure ventilation (NIPPV) is recommended as first‐line therapy, with LISA preferentially used for spontaneously breathing infants who require surfactant therapy.

The physiologic rationale for combining sNIPPV with LISA is compelling: surfactant administration causes rapid changes in lung compliance, and synchronized mechanical support during this critical period may optimize gas exchange and minimize acute destabilization. This review examines the individual and combined clinical applications of these two modalities.

## 2 Physiologic Rationale

### 2.1 LISA

LISA administers surfactant through a thin catheter, maintaining spontaneous ventilation and noninvasive respiratory support. Compared with the traditional intubation, surfactant administration, and extubation (INSURE) approach, LISA offers several advantages:

- Reducing the need for mechanical ventilation within 72 hours of administration (relative risk of approximately 0.66) [2, 3].
- Lowering the risk of bronchopulmonary dysplasia (BPD) [2].
- Reducing the risk of severe intraventricular hemorrhage [4].
- Minimizing hemodynamic instability and volutrauma

The maintenance of spontaneous breathing with pressure support preserves functional residual capacity (FRC) and facilitates more uniform surfactant distribution throughout the lung.

### 2.2 sNIPPV

sNIPPV delivers synchronized positive pressure breaths through various synchronization mechanisms—flow‐sensing, Graseby technology, or electrical diaphragm activity (Edi) signals. When compared with conventional CPAP:

- sNIPPV improves thoraco‐abdominal synchrony and reduces the work of breathing by matching patient effort [5, 6].
- It decreases apnea and bradycardia episodes [7].
- It increases FRC and improves tidal volume [8].
- It reduces extubation failure by approximately 30% compared with CPAP as confirmed by multiple meta‐analyses [9].

### 2.3 Rationale for Combining sNIPPV With LISA

Surfactant administration frequently results in abrupt improvements in lung compliance. Furthermore, during intubation for surfactant administration, there is loss of positive pressure thereby loss of FRC and alveolar recruitment, a problem which can be overcome by LISA. sNIPPV has also shown to decrease the risk of respiratory failure and to have a trend toward decreasing BPD when compared to CPAP on its own. Hence, when both these modalities LISA and sNIPPV are used in combination, they may be beneficial by:

- Augmenting ventilation: Providing additional tidal volume during periods when spontaneous breathing effort is insufficient
- Enhanced gas exchange: Improving CO_2_ clearance and oxygenation stability
- Apnea management: Reducing frequency of apnea‐related hypoxemic episodes through mandatory breaths
- Hemodynamic support: Maintaining more stable intrathoracic pressures during and immediately after LISA
- FRC maintained: Having better alveolar recruitment and continuous positive pressure during LISA.

## 3 Clinical Evidence

### 3.1 LISA Versus INSURE

Several randomized trials and meta‐analyses consistently show that LISA provides clear clinical advantages over the INSURE technique. INSURE is repeatedly associated with higher rates of procedure‐related desaturation, bradycardia, and early mechanical ventilation. In a randomized trial published in JAMA Pediatrics, Humberg et al. [4] reported that LISA significantly reduced the need for mechanical ventilation within 72 hours (odds ratio [OR] ≈ 0.56, corresponding to a reduction about 44%) and decreased the combined outcome of BPD or death by nearly 20% compared with INSURE. Observational cohorts similarly demonstrate 5%–12% lower BPD incidence in infants managed with LISA. Although a study by Permall et al. [10] did not find statistically significant differences in BPD rates, infants receiving LISA under sNIPPV showed better FiO_2_ stability, fewer desaturation episodes, and milder early respiratory severity, reinforcing broader evidence that LISA is physiologically more stable and clinically safer than INSURE [9, 11].

### 3.2 sNIPPV Alone

Evidence for sNIPPV is strong across several systematic reviews. A Cochrane systematic review and meta‐analysis found that NIPPV reduces extubation failure by about 30% (risk ratio [RR] ≈ 0.70) compared with CPAP alone [12]. Additional analysis [13] indicated that synchronized modes provide the most consistent improvements, with up to 40% fewer apnea‐related desaturations and better minute ventilation than unsynchronized NIPPV. Studies, such as the meta‐analysis by Isayama et al. [14], also showed that NIPPV reduces the need for mechanical ventilation (OR ≈ 0.78). Together, these results position sNIPPV as a superior noninvasive support modality particularly valuable during surfactant procedures where respiratory stability is critical.

### 3.3 Role of sNIPPV as Primary and Secondary Mode of Respiratory Support

#### 3.3.1 NIPPV/sNIPPV as Primary Mode (Initial Respiratory Support)

Meta‐analysis data summarized by Deguise and Lemyre et al. [15] showed that NIPPV is superior to CPAP as primary support, reducing respiratory failure (RR 0.65, 95% confidence interval [CI] 0.54–0.78) with a number needed to treat (NNT) of 13. Ventilator‐generated and sNIPPV provide the strongest benefit. Importantly, primary‐use NIPPV is associated with a significant reduction in BPD (RR 0.70, 95% CI 0.52–0.92), an effect not seen consistently in postextubation use. These findings suggest that NIPPV—especially sNIPPV—offers the greatest advantage when used early in the respiratory course.

#### 3.3.2 NIPPV/sNIPPV as Secondary/Post‐Extubation Mode

As a rescue mode after extubation, NIPPV reduces the risk of re‐intubation, though the effect is smaller than that used in the primary mode. The Cochrane update reported reduced extubation failure (RR 0.75, 95% CI 0.67–0.84) with an NNT of 11. However, unlike primary support, postextubation NIPPV was not shown to significantly lower BPD rates (RR 0.93, 95% CI 0.84–1.05). Although sNIPPV shows a trend toward decreasing BPD, this effect was not as significant compared to using sNIPPV in the primary mode. These physiologic and clinical advantages position sNIPPV as an effective rescue therapy that may indirectly contribute to improved long‐term pulmonary outcomes.

### 3.4 Studies Evaluating the Combination

Clinical evidence for combined sNIPPV–LISA therapy is emerging but increasingly supportive. The Permall et al. (2024) study [10], published in Scientific Reports, provides the most recent direct evaluation of this combination. This prospective cohort study compared outcomes between infants receiving LISA with sNIPPV support versus those managed with traditional INSURE with CPAP support (Table 1). The key findings included the following:

- The reductions in FiO_2_ requirements were greater and more sustained in the sNIPPV–LISA group.
- There were significantly fewer desaturation and bradycardia episodes during and after the procedure in the sNIPPV‐LISA group.
- There was a numerical trend toward lower re‐intubation rates in the first 7 days of life in the sNIPPV‐LISA group (12% vs. 18% between the CPAP–LISA comparison group).
- The sNIPPV–LISA group demonstrated fewer cases of BPD compared to CPAP–INSURE group (10 vs. 21, 31.3% vs. 36.8%, and *p* > 0.05), but the difference was not statistically significant. There was no difference in mortality rates between the two groups.

**TABLE 1:** sNIPPV–LISA versus CPAP–INSURE outcomes in preterm with RDS.

Outcome	sNIPPV–LISA	CPAP–INSURE	sNIPPV–LISA advantage
Mechanical ventilation within 72 h [9, 10]	RR 0.60 (95% CI 0.47–0.76)	Baseline	40% reduction
BPD rates [2, 9, 10]	RR 0.76; 31%	37%	Trend to be lower (*p* > 0.05)
Re‐intubation (7 days) [10]	12%	18%	Lower trend
Desaturation episodes [10]	Fewer	More	Significantly fewer
FiO_2_ stability [10]	Better	Standard	Superior
Procedural stability [10]	Excellent	Standard	Enhanced
Apnea frequency [5, 12]	Reduced 30%–40%	Higher	Substantially lower
Safety profile	Mild adverse effects	Standard	Comparable

Abbreviations: BPD, bronchopulmonary dysplasia; CPAP, continuous positive airway pressure; INSURE, intubation, surfactant administration, and extubation; LISA, less‐invasive surfactant administration; sNIPPV, synchronized nasal intermittent positive pressure ventilation.

Clinical experience reports from multiple neonatal intensive care units (NICUs) implementing combined sNIPPV–LISA as standard practice for eligible infants have reported favorable observations, including lower re‐intubation rates during the first 72 hours of life and fewer hypoxemic episodes requiring intervention. Although these reports are observational and lack the rigor of randomized trials, they provide encouraging preliminary evidence that the combined approach may offer practical clinical advantages in routine care settings [14].

## 4 Practical Considerations

According to our unit protocol, commonly used initial sNIPPV settings are PEEP 5–6 cmH_2_O, PIP 16–20 cmH_2_O, rate 30–40 breaths per minute, and inspiratory time of 0.35–0.5 seconds. LISA should be performed under sNIPPV support using a thin catheter, warmed surfactant, and without suctioning afterward. Complications, such as nasal trauma or transient bradycardia, are generally mild [7].

## 5 Conclusion

The combined use of sNIPPV with LISA represents a physiologically coherent and increasingly evidence‐supported strategy for managing RDS in preterm infants. LISA has been associated with reductions in BPD or BPD/death in some randomized trials; however, consistent benefits for BPD have not yet been established. When applied together, early clinical studies, including the 2024 cohort by Permall et al. [10], demonstrate improved procedural stability, fewer desaturations, better FiO_2_ trajectories, trends toward lower re‐intubation rates, and trends toward decreasing BPD rates when used in the early stages (primary mode), although consistent benefits for BPD have not yet been established. Overall, emerging data suggest that the sNIPPV–LISA may offer additive advantages in the respiratory management of preterm babies compared to existing therapies. Larger well‐powered randomized trials and meta‐analyses are needed to clarify long‐term pulmonary outcomes, but current evidence supports this combination as a promising evolution in noninvasive neonatal respiratory care.

## Author Contributions

**Aniket P. Singh:** conceptualization, literature review, manuscript drafting, critical revision, and final approval of the manuscript. **Raghuram Mallaiah:** conceptualization, critical revision of the manuscript, supervision, and final approval of the manuscript.

## Funding

The authors have nothing to report.

## Conflicts of Interest

The authors declare no conflicts of interest.

## Data Availability

Data sharing is not applicable to this article as no datasets were generated or analyzed during the current study.
